# Financial toxicity in sarcoma patients and survivors in Germany: results from the multicenter PROSa study

**DOI:** 10.1007/s00520-021-06406-3

**Published:** 2021-07-11

**Authors:** Matthias Büttner, Susanne Singer, Leopold Hentschel, Stephan Richter, Peter Hohenberger, Bernd Kasper, Dimosthenis Andreou, Daniel Pink, Kathy Taylor, Karin Arndt, Martin Bornhäuser, Jochen Schmitt, Markus K. Schuler, Martin Eichler

**Affiliations:** 1 Division of Epidemiology and Health Services Research, Institute of Medical Biostatistics, Epidemiology and Informatics (IMBEI), University Medical Center Mainz, Obere Zahlbacher Straße 69, 55131 Mainz, Germany; 2 University Cancer Centre, Mainz, Germany; 3 National Center for Tumor Diseases (NCT/UCC), Dresden, Germany; 4 Clinic and Polyclinic for Internal Medicine I, University Hospital Carl Gustav Carus, TU Dresden, Dresden, Germany; 5 Division of Surgical Oncology & Thoracic Surgery, Mannheim University Medical Center, University of Heidelberg, Mannheim, Germany; 6 Sarcoma Unit, Interdisciplinary Tumor Center, University Medical Center Mannheim, Mannheim, Germany; 7 Department of General Orthopedics and Tumor Orthopedics, University Hospital Munster, Münster, Germany; 8 Sarcoma Center Berlin-Brandenburg, Helios Hospital Bad Saarow, Bad Saarow, Germany; 9 Department of Internal Medicine C, University Hospital Greifswald, Greifswald, Germany; 10 German Sarcoma Foundation, Woelfersheim, Germany; 11 Center for Evidence-Based Healthcare, University Hospital Carl Gustav Carus, Technical University Dresden, Dresden, Germany

**Keywords:** Sarcoma, Financial toxicity, Burden, Cancer, Germany, Financial difficulties

## Abstract

**Purpose:**

Cancer patients have been shown to frequently suffer from financial burden before, during, and after treatment. However, the financial toxicity of patients with sarcoma has seldom been assessed. Therefore, the aim of this study was to evaluate whether financial toxicity is a problem for sarcoma patients in Germany and identify associated risk factors.

**Methods:**

Patients for this analysis were obtained from a multicenter prospective cohort study conducted in Germany. Using the financial difficulties scale of the EORTC QLQ-C30, financial toxicity was considered to be present if the score exceeded a pre-defined threshold for clinical importance. Comparisons to an age- and sex-matched norm population were performed. A multivariate logistic regression using stepwise backward selection was used to identify factors associated with financial toxicity.

**Results:**

We included 1103 sarcoma patients treated in 39 centers and clinics; 498 (44.7%) patients reported financial toxicity. Sarcoma patients had 2.5 times the odds of reporting financial difficulties compared to an age- and sex-matched norm population. Patient age < 40 and > 52.5 years, higher education status, higher income, and disease progression (compared to patients with complete remission) were associated with lower odds of reporting financial toxicity. Receiving a disability pension, being currently on sick leave, and having a disability pass were statistically significantly associated with higher odds of reporting financial toxicity.

**Conclusion:**

Financial toxicity is present in about half of German sarcoma patients, making it a relevant quality of life topic for patients and decision-makers.

## Introduction

Sarcomas are a rare group of cancers of mesenchymal origin and are estimated to represent 1 to 2% of adult cancers worldwide [1]. The 5-year overall survival probability amounts to approximately 60% and depends on a variety of risk factors [2]. Quality of life impairments among sarcoma patients have been reported across all sarcoma subgroups and are present during diagnosis, treatment, and follow-up [3].

One important aspect of quality of life that has gained increasing awareness within the last years is the financial burden of cancer patients. The financial burden of cancer refers not only to out-of-pocket payments (OOPPs) and income losses, but also material consequences and psychological effects or behavioral changes that are then expressed as financial toxicity [4–7]. It has been shown that patients reporting financial difficulties may have an increased risk of distress, anxiety, and depression [8, 9], show impairments in quality of life [8–11], and may even have worse survival [12]. This problem is not only observed in countries with high OOPPs, like the USA, but also in countries with universal health coverage [13–16].

Financial toxicity studies are often conducted in general cancer populations or are performed for the most common cancers, such as lung, breast, or colon cancer [13–19]. To our knowledge, studies evaluating financial toxicity in sarcoma patients in detail have not been performed. It has, however, been shown that the financial impact of the disease and the resulting financial toxicity is an important and relevant topic for patients with sarcoma [17]. Additionally, patients can be diagnosed with sarcoma throughout the whole course of life [1, 2] and therefore also during their social, educational, and occupational development and this might also influence their financial abilities throughout the rest of their life.

Therefore, the aim of this study was to evaluate the extent of financial toxicity in German sarcoma patients and survivors in Germany and identify possible risk factors.

## Methods

### Design

Patients for this analysis were obtained from the PROSa study, which is a multicenter prospective cohort study conducted in Germany with two follow-ups at 6 and 12 months after baseline. Follow-up was defined as a visit to one of the participating study centers [18]. The baseline data used for this analysis were obtained between 09/2017 and 02/2019. Inclusion criteria for the PROSa study were a confirmed diagnosis of any type of sarcoma regardless of the current stage of the disease course (at diagnosis, in treatment, or in follow-up) and the ability to give written informed consent. Patients who were not able to fill out questionnaires due to linguistic reasons (restricted to German language) or cognitive impairment were excluded from the study. Ethical approval was obtained by the ethics committee of the Technical University of Dresden (AZ: EK 1,790,422,017) and the ethics committees of the participating centers. A more detailed description of the PROSa study can be found in [18].

### Assessments

Clinical data of the participants were obtained using case report forms through the participating medical centers. Aftercare was defined as “yes” for patients with being in after care or for having completed their treatment. Sociodemographic data and patient reported outcomes (PROs) were directly obtained from the patient by letter or online questionnaires. The following socioeconomic and sociodemographic variables were used for the analysis: age, sex, education, occupation, employment status, income, sick leave status, and whether a patient had a disability pass. Education was defined by the highest educational certificate attained divided into the following categories: none to secondary (8/9 years), secondary school (10 years), vocational baccalaureate, high school/baccalaureate, and other/unknown. Occupation was assessed by the classification of labor type (e.g., blue-collar worker, civil servant) and employment status was determined by the employment setting (e.g., self-employed, retired, etc.). Income was assessed using the OECD equivalence scale [19] as a continuous variable but also categorized into income groups ≤ €1250, €1251 to €1750, €1751 to €2250, €2251 to €2750, > €2750, and unknown.

For the assessment of financial toxicity, the financial difficulties scale of the EORTC QLQ-C30 was used [20]. This 1-item scale measures whether the physical conditions or the medical treatment has caused any financial difficulties within the last week using a four-point Likert scale (“not at all,” “a little,” “quite a bit,” and “very much”). The answer is converted to a scale ranging from 0 to 100, with high values indicating a high financial burden perceived by the patient in accordance with the scoring manual of the EORTC [21]. Patients were considered to have financial toxicity if their score in the financial difficulties scale was above the threshold for clinical importance (17 points) identified by Giesinger et al. [22]. Patients with a score above 17 points at least report any financial difficulties (“a little,” “quite a bit,” or “very much”).

### Statistical analysis

The analysis was performed on a cross-sectional basis. Patient characteristics are expressed as mean values or percentages according to the data. Univariate comparisons between patients with financial toxicity and patients without were conducted using Chi-square tests. Financial difficulty scores of all sarcoma patients, curative sarcoma patients, and palliative sarcoma patients were compared to a norm population [23]. Multivariate logistic regression with backward stepwise selection with p > 0.1 as the exclusion criterion was performed with reporting financial difficulties (yes/no) as the dependent variable and clinical and socioeconomic characteristics as independent variables (Table 1). Variables were included in regression after being tested for non-multicollinearity (correlation < 0.8). All statistical analyses were performed using IBM SPSS Statistics version 25.0 (Armonk, NY).

**Table 1:** Description of baseline characteristics

Variable	Value	All N = 1103 (column%)	Financial toxicity—yes N = 498 (44.7%)	Financial toxicity—no N = 605 (54.9%)
Socioeconomic factors				
Sex	Female	537 (48.7)	243 (48.8)	294 (48.7)
	Male	565 (51.3)	255 (51.2)	310 (51.3)
Age at study inclusion*,**	Mean	56.6 (SD 15.8)	54.5 (SD 14.5)	58.4 (SD 16.6)
	Median IQR	58.4 (47.6; 68.5)	55.4 (46.6; 63.6)	61.6 (49.8; 70.6)
Age at study inclusion—group*	18– < 27.5 years	78 (7.1)	33 (6.7)	45 (7.4)
	27.5– < 40 year	104 (9.4)	44 (8.9)	60 (9.9)
	40– < 52.5 years	194 (17.6)	121 (24.4)	73 (12.1)
	52.5– < 65 years	366 (33.2)	194 (39.1)	172 (28.4)
	65– < 77.5 years	277 (25.2)	81 (16.3)	196 (32.4)
	> 77.5 years	82 (7.4)	23 (4.6)	59 (9.8)
Household size (living alone)#	No	887 (80.4)	400 (80.3)	487 (80.5)
	Yes	206 (18.7)	93 (18.7)	113 (18.7)
	Unknown	10 (0.9)	5 (1.0)	5 (0.8)
Children in household#	No	884 (80.1)	392 (78.7)	492 (81.3)
	Yes	151 (13.7)	73 (14.7)	78 (12.9)
	Unknown	68 (6.2)	33 (6.6)	35 (5.8)
School leaving certificate*,#	None to secondary school (8/9 years)	264 (23.9)	143 (28.7)	121 (20.0)
	Secondary school (10 years)	376 (34.1)	177 (35.5)	199 (32.9)
	Vocational baccalaureate	120 (10.9)	51 (10.2)	69 (11.4)
	High school/baccalaureate	317 (28.7)	115 (23.1)	202 (33.4)
	Something else/unknown	26 (2.4)	12 (2.4)	14 (2.3)
Occupational status*,#	Blue-collar worker	208 (18.9)	118 (23.7)	90 (14.9)
	Civil servant (1)	81 (7.3)	23 (4.6)	58 (9.6)
	White collar worker (2)	611 (55.4)	266 (53.4)	345 (57.0)
	Self-employed (3)	104 (9.4)	49 (9.8)	55 (9.1)
	Unknown/not applicable (4)	99 (9.0)	42 (8.4)	57 (9.4)
Equivalent income*,**	Mean (standard deviation)	2047€ (1105€)	1800€ (1010€)	2251€ (1138€)
Equivalized income*,#	≤ 1250	231 (20.9)	135 (27.1)	96 (15.9)
	1250.1–1750	213 (19.3)	115 (23.1)	98 (16.2)
	1750.1–2250	247 (22.4)	103 (20.7)	144 (23.8)
	2250.1–2750	89 (8.1)	26 (5.2)	63 (10.4)
	≥ 2750.1	184 (16.7)	58 (11.6)	126 (20.8)
	Unknown	139 (12.6)	61 (12.2)	78 (12.9)
Employment status*,#	Employed/self-employed	490 (44.4)	226 (45.4)	264 (43.6)
	Unemployed	45 (4.1)	31 (6.2)	14 (2.3)
	Disability pension	138 (12.5)	103 (20.7)	35 (5.8)
	Early retirement/retirement pension/partial retirement	376 (34.1)	114 (22.9)	262 (43.3)
	Housewife/houseman	26 (2.4)	11 (2.2)	15 (2.5)
	School, apprenticeship, study	17 (1.5)	8 (1.6)	9 (1.5)
	Unknown	11 (1.0)	5 (1.0)	6 (1.0)
Sick leave*,#	No	822 (74.5)	327 (65.7)	495 (81.8)
	Yes	257 (23.3)	163 (32.7)	94 (15.5)
	Unknown	24 (2.2)	8 (1.6)	16 (2.6)
Disabled person pass*,#	No	405 (36.7)	131 (26.3)	274 (45.3)
	Yes	689 (62.5)	364 (73.1)	325 (53.7)
	Unknown	9 (0.8)	3 (0.6)	6 (1.0)
Clinical factors				
Time since diagnosis#	0– < 0.5 years	209 (19.0)	95 (19.1)	114 (18.9)
	0.5– < 1 year	126 (11.4)	67 (13.5)	59 (9.8)
	1– < 2 years	165 (15.0)	79 (15.9)	86 (14.2)
	2– < 5 years	290 (26.3)	131 (26.4)	159 (26.3)
	More than 5 years	311 (28.2)	125 (25.2)	186 (30.8)
Sarcoma type—detail§	Liposarcoma	210 (19.1)	94 (19.0)	116 (19.2)
	Unclassified sarcoma (1)	163 (14.8)	68 (13.7)	95 (15.7)
	Fibroblastic, myofibroblastic, fibrohistiocytic sarcoma (2)	130 (11.8)	52 (10.5)	78 (12.9)
	GIST (3)	130 (11.8)	51 (10.3)	79 (13.0)
	Leiomyosarcoma (4)	131 (11.9)	66 (13.3)	65 (10.8)
	Osteosarcoma (5)	71 (6.5)	30 (6.0)	41 (6.8)
	Chondrosarcoma (6)	63 (5.7)	32 (6.5)	31 (5.1)
	Ewing sarcoma (7)	43 (3.9)	28 (5.6)	15 (2.5)
	Synovial sarcoma (8)	47 (4.3)	24 (4.8)	23 (3.8)
	Other (9)	112 (10.2)	51 (10.3)	61 (10.1)
Site§	Trunk	518 (47.1)	231 (44.6)	287 (47.5)
	Limbs	522 (47.5)	234 (47.1)	288 (47.7)
	Somewhere else	60 (5.5)	31 (6.3)	29 (4.8)
Grading§	Low grade	137 (12.4)	52 (10.4)	85 (14.0)
	High grade	598 (54.2)	283 (56.8)	315 (52.1)
	Not applicable/unknown	368 (33.4)	163 (32.7)	205 (33.9)
T-Stage§	T1	171 (15.5)	76 (15.3)	95 (15.7)
	T2–T4	516 (46.8)	232 (46.6)	284 (46.9)
	Other/unknown	416 (37.7)	190 (38.2)	226 (37.4)
Malignancy of tumor*,§	Locally aggressive + rarely metastatic	86 (7.8)	28 (5.6)	58 (9.6)
	Malignant	1014 (92.2)	468 (94.4)	546 (90.4)
Metastasis until time of study inclusion*	No metastasis	604 (54.8)	252 (50.6)	352 (58.2)
	Metastasis	365 (33.1)	180 (36.1)	185 (30.6)
	Unknown	134 (12.1)	66 (13.3)	68 (11.2)
Tumor recurrence***,#	No recurrence	794 (72.0)	355 (71.3)	439 (72.6)
	Recurrence	278 (25.2)	127 (25.5)	151 (25.0)
	Suspicion	12 (1.1)	7 (1.4)	5 (0.8)
	Unknown	19 (1.7)	9 (1.8)	10 (1.7)
Treatment intention**,#	Curative	820 (74.6)	359 (72.5)	461 (76.3)
	Palliative	258 (49.6)	128 (25.9)	130 (21.5)
	Unknown	21 (1.9)	8 (4.2)	13 (2.2)
Disease status#	Complete remission	491 (44.5)	219 (44.0)	272 (45.0)
	Partial remission/stable disease	326 (29.6)	148 (29.7)	178 (29.4)
	Progressive	160 (14.5)	68 (13.7)	92 (15.2)
	Unknown	126 (11.4)	63 (12.7)	63 (10.4)
Aftercare status*,#	Not in aftercare	468 (42.4)	231 (46.4)	237 (39.2)
	In aftercare	620 (56.2)	262 (52.6)	358 (59.2)
	Unknown	15 (1.2)	5 (1.0)	10 (1.7)
Surgery***,#	No	126 (11.4)	59 (11.8)	67 (11.1)
	Yes	969 (87.9)	435 (87.3)	534 (88.3)
	Unknown	8 (0.7)	4 (0.8)	4 (0.7)
Chemotherapy*,***,#	No	568 (51,5)	228 (45.8)	340 (56.2)
	Yes	523 (47.4)	264 (53.1)	259 (42.8)
	Unknown	12 (1.1)	6 (1.2)	6 (1.0)
Radiotherapy***,#	No	646 (58.6)	285 (57.2)	361 (59.7)
	Yes	430 (39.0)	201 (40.4)	229 (37.9)
	Unknown	27 (2.4)	12 (2.4)	15 (2.5)
Treatment lines#	No treatment yet (1)	26 (2.4)	9 (1.8)	17 (2.8)
	One treatment line	491 (44.5)	216 (43.4)	275 (45.5)
	More than one treatment line (2)	547 (49.6)	257 (51.6)	290 (47.9)
	Unknown (3)	39 (3.5)	16 (3.2)	23 (3.8)

^*^Chi-square p < 0.05

^**^Variable not in the multivariate model

^***^Model variable adapted

^#^At/until time of study inclusion

^§^At time of diagnosis

## Results

### Sample and patient characteristics

In total, 1309 sarcoma patients from 39 sarcoma centers and clinics participated in the study, of whom 1103 (84.3%) provided data regarding financial toxicity. The mean age of the study population at recruitment was 52.6 years, and 48.7% of the participants were female. Further sociodemographic and clinical characteristics can be found in Table 1.

### Financial toxicity in German sarcoma patients

A total of 498 (44.7%) of the sarcoma patients reported suffering from financial difficulties above the threshold for clinical importance. Differences between patients with and without financial toxicity are presented in Table 1. Statistically significant differences between the two groups were seen for age, education, occupation, income, employment, disability pass, biological behavior of the tumor, metastasis at baseline, aftercare status, and chemotherapy.

**Fig. 1:**
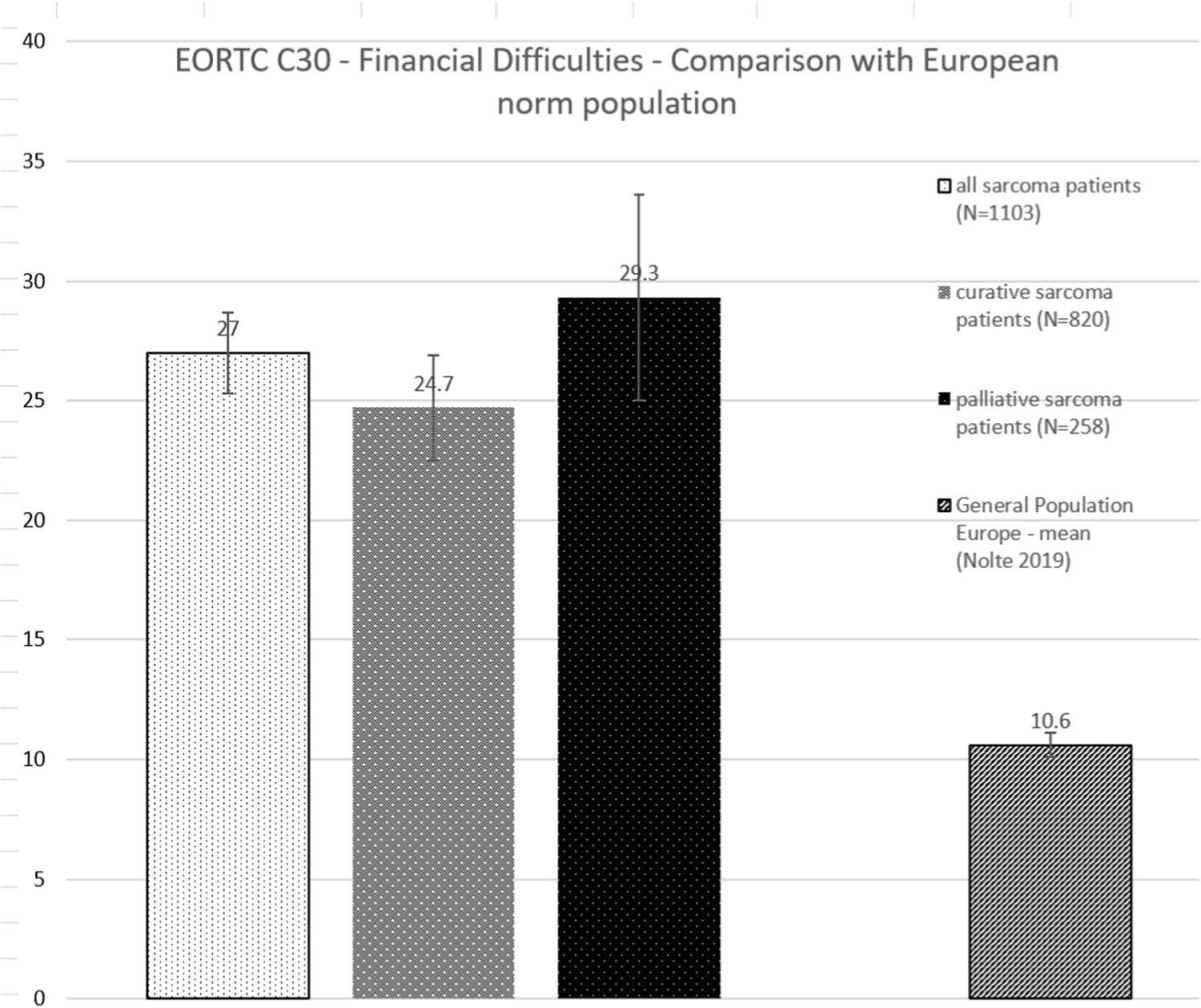
Comparison of financial difficulties scale to an age- and sex-adjusted norm population

Compared to the German norm population [23], sarcoma patients in the study had statistically higher scores, indicating more problems in the financial difficulties scale; this difference also persisted after stratification into curative and palliative sarcoma patients (Fig. 1). All sarcoma patients had 2.5 times the odds of reporting financial difficulties compared to the age- and sex-matched norm population.

### Variables associated with financial toxicity in German sarcoma patients

The final variable selection of the multivariate logistic regression with backward stepwise selection and the respective results can be found in Table 2. Compared to the age group from 40 to < 52.5 years, younger (18 to < 27.5 years (OR: 0.45; 95% CI [0.23;0.88]), 27.5 to < 40 years (OR: 0.54; 95% CI [0.31;0.94])) and older (52.5 to < 65 years (OR: 0.66; 95% CI [0.44;0.99]), 65 to < 77.5 years (OR: 0.25; 95% CI [0.12;0.49]), and > 77.5 years (OR: 0.27; 95% CI [0.12;0.62])) age groups had statistically lower odds of reporting financial toxicity. Higher education (compared to none to secondary school; secondary school (OR: 0.57; 95% CI [0.39;0.84]), vocational baccalaureate (OR: 0.67; 95% CI [0.39;1.15]), high school/baccalaureate (OR: 0.51; 95% CI [0.33;0.79])) and higher income (compared to ≤ €1250; €1250.1 to €1750 (OR: 0.93; 95% CI [0.60;1.43]), €1750.1 to €2250 (OR: 0.47; 95% CI [0.30;0.72]), €2250.1 to €2750 (OR: 0.24; 95% CI [0.13;0.45]), ≥ €2750.1 (OR0.33; 95% CI [0.12;0.53])) were also associated with having lower chances of reporting financial toxicity. Receiving a disability pension (OR: 3.29; 95% CI [1.95;5.54], compared to employed/self-employed), being currently on sick leave (OR: 2.40; 95% CI [1.60;3.62]), compared to not being currently on sick leave), and having a disability pass (OR: 2.52; 95% CI [1.81;3.49]) were statistically significantly associated with higher odds of reporting financial toxicity. Patients more than 5 years post diagnosis (compared to 0 to < 0.5 years since diagnosis) reported statistically significant lower odds (OR: 0.58; 95% CI [0.35;0.94]) of reporting financial toxicity. Compared to patients with complete remission, patients with progressive disease had statistically significant lower odds (OR: 0.48; 95% CI [0.30;0.77]) of reporting financial toxicity, while for patients with partial remission/stable disease no differences in odds (OR: 0.96; 95% CI [0.67;1.37]) were observed.

**Table 2:** Results of the multivariate logistic regression using stepwise backward selection with having financial difficulties as the dependent variable (final model)

Independent variables	OR [95% CI]
Age at study inclusion (p = 0.002)	
18 to < 27.5 years	0.45 [0.23;0.88]
27.5 to < 40 years	0.54 [0.31;0.94]
40 to < 52.5 years (reference category)	1
52.5 to < 65 years	0.66 [0.44;0.99]
65 to < 77.5 years	0.25 [0.12;0.49]
> 77.5 years	0.27 [0.12;0.62]
Education (p = 0.023)	
None to secondary school (8/9 years) (ref.)	1
Secondary school (10 years)	0.57 [0.39;0.84]
Vocational baccalaureate	0.67 [0.39;1.15]
High school/baccalaureate	0.51 [0.33;0.79]
Something else/unknown	0.98 [0.37;2.60]
Occupational status (p = 0.081)	
Blue-collar worker (ref.)	1
Civil servant	1.01 [0.52;1.98]
White collar worker	0.95 [0.64;1.41]
Self-employed	1.85 [1.04;3.27]
Unknown/not applicable	0.76 [0.41;1.41]
Equivalent income (p < 0.001)	
≤ €1250 (ref.)	1
€1250.1 to €1750	0.93 [0.60;1.43]
€1750.1 to €2250	0.47 [0.30;0.72]
€2250.1 to €2750	0.24 [0.13;0.45]
≥ €2750.1	0.33 [0.12;0.53]
Unknown	0.58 [0.35;0.94]
Employment status (p = 0.002)	
Employed/self-employed (ref.)	1
Unemployed	1.58 [0.75;3.31]
Disability pension	3.29 [1.95;5.54]
Early retirement/retirement pension/partial retirement	1.13 [0.61;2.11]
Housewife/houseman	1.40 [0.54;3.62]
School, apprenticeship, study	1.24 [0.38;4.10]
Unknown	1.34 [0.30;6.02]
Sick leave (p < 0.001)	
No (ref.)	1
Yes	2.40 [1.60;3.62]
Unknown	1.12 [0.37;3.39]
Disabled person pass (p < 0.001)	
No (ref.)	1
Yes	2.52 [1.81;3.49]
Unknown	1.30 [0.21;8.03]
Time since diagnosis (0 to < 0.5 years) (p = 0.024)	
0 to < 0.5 years (ref.)	1
0.5 to < 1 year	1.31 [0.77;2.22]
1 to < 2 years	0.87 [0.52;1.47]
2 to < 5 years	0.83 [0.51;1.36]
More than 5 years	0.58 [0.35;0.94]
Metastasis until baseline (p = 0.054)	
No metastasis (ref.)	1
Metastasis	1.38 [0.97;1.97]
Unknown	1.56 [1.01;2.41]
Disease status (p = 0.010)	
Complete remission (ref.)	1
Partial remission/stable disease	0.96 [0.67;1.37]
Progressive	0.48 [0.30;0.77]
Unknown	1.11 [0.68;1.82]

Variables included in the full model before backward selection: sex, age at study inclusion, household size, children in household, school leaving certificate, occupational status, equivalized income, employment status, sick leave, disabled person pass, time since diagnosis, sarcoma type, site, grading, T-Stage, malignancy of tumor, metastasis until baseline, tumor recurrence, disease status, aftercare status, treatment lines, and treatment received

## Discussion

Our analysis showed that sarcoma patients suffer from financial difficulties in a universal health coverage system like Germany’s. 44.7% of the patients reported financial difficulties measured by the EORTC QLQ-C30, and 2.5 times the odds of reporting financial toxicity compared to an age- and sex matched norm population was observed.

These impairments regarding financial difficulties are in line with other studies using the financial difficulties scale of the QLQ-C30 in sarcoma patients [24–28]. Hudgens et al. [24] reported a financial difficulties score of 25.0 (SD: 29.5) at baseline and 29.5 (SD: 34.2) at progression in a study consisting of 452 sarcoma patients with advanced, progressing disease. A longitudinal study by Paredes et al. [25] with 36 sarcoma patients found higher financial difficulties scores compared to a norm population in the diagnostic phase and 4 months after initiation of treatment.

Financial difficulties are not restricted to sarcoma patients and have been observed in other cancer populations across different health care settings [13–16]. Financial toxicity often results from OOPPs, paid by the patient for the treatment of the disease and related care and from income changes. OOPPs in sarcoma patients alone have not been evaluated and were not assessed in our study. For other cancer entities and mixed cancer populations, it has been reported that OOPPs occur at a high level in countries with higher self-payments [29] but are also present at a moderate level in countries with universal health insurance [14, 30, 31]. For a German mixed cancer population, Büttner et al. [14] found average 3-month OOPPs of €205 at the end of hospital stay, and €180 and €148 at 3 and 15 months after their hospital stay.

Income losses may also have an impact on financial difficulties in cancer patients. In our study, regression analysis showed that higher income was significantly associated with lower chances of reporting financial toxicity. This is also in line with previous studies in other cancer populations [32]. Changes in income are often related to changes in one’s ability to work. Approximately 60–70% of all cancer patients return to work [15, 33], with the rest going into retirement or applying for disability pensions. Patients with sarcoma show the lowest return to work rates [34] and are considered to have a higher risk of not returning to work [35, 36]. A study by Laros et al. [37] showed that sarcoma patients in Germany had an average unemployment rate of 8.8 months and that for 67% of the sarcoma patients, the work situation changed after the unemployment phase. Patients in Germany are allowed up to 72 weeks of sick leave before returning to work or applying for a pension. In our study, 23.3% of the patients were currently on sick leave, and sick leave was associated with a higher chance for reporting financial difficulties. Hazell et al. [38] found a similar association of sick leave and financial toxicity in lung cancer patients in the USA. Patients on sick leave in Germany receive 70% of their gross salary from their health insurance [39]. This income reduction in combination with after treatment OOPPs [14] might lead to financial problems or financial insecurity.

Another possible factor for financial difficulties during sick leave might be the reduction of income due to high surcharges not covered by the insurance [40]. If patients are not able to return to work after sick leave, it is mandatory to apply for a disability pension in Germany. The average full disability pension, which applies if you are not able to work more than 3 h a day, was €850 in 2019 [41], indicating a severe income decline for patients who were fully working before their disease. Laros et al. [37] found an income loss of 62% on average for sarcoma patients in Germany who applied for a pension or partial or total unemployment benefits. The effect of this income decline is expressed in a statistically significantly increased chance of financial toxicity for patients receiving disability pension in our analysis, where having a disability pass was associated with higher odds of reporting financial toxicity. This might seem controversial because a disability pass may lead to deductions in certain services or payments (e.g., entrance fees, transportation tickets) [40]. On the other hand, patients with a disability pass might have higher OOPPs due to a need for additional services or medical aids which are not fully covered by their insurance.

Higher education was associated with lower chances of financial toxicity in our study, which is in line with findings for other cancer populations [32]. Our analysis has shown that, compared to the age group 40 to < 52.5 years, younger and older age groups reported lower chances for reporting financial toxicity. We can only speculate as to possible reasons for this finding. Older patients may have acquired higher financial reserves during their lifetime, while our reference age group might be at a disadvantage due to financial investments, such as loans or substantial purchases. Additionally, older patients are already retired, and the income losses due to the disease may not have as great an impact because they have a stable income from their pension fund. Younger patients, on the other hand, may not yet have substantial financial investments, which might explain the lower financial toxicity reported. Furthermore, their baseline income might be lower, so that the resulting difference to the disability pension sum might be less pronounced compared to older patients. Nevertheless, studies in other cancer patients have reported that younger age is more often associated with financial toxicity [32].

Another surprising finding of our study was that patients with progressive disease had lower odds of reporting financial toxicity compared to patients with complete remission. The association of disease status and financial toxicity has been rarely assessed in the literature. Zafar et al. [42] found no statistically significant association between patients with recurrent or progressive cancer and financial distress. On the other hand, Walker et al. [43] reported more financial difficulties in patients with disease progression for advanced non-squamous NSCLC. A Finish study examining the OOPPs of breast, prostate, and colorectal cancer patients reported the lowest 6-month OOPPs for patients in remission (€224) compared to palliative patients (€603), patients with metastatic disease (€383), in rehabilitation (€264), and in primary treatment (€263) [44]. Future studies should address this issue in sarcoma patients and further evaluate possible reasons, provided that our findings can be validated in other sarcoma patient cohorts.

Patients with more than 5 years past diagnosis had lower chances of reporting financial toxicity compared to patients with 0– < 0.5 years past diagnosis. Sarcoma patients currently receiving treatment have been shown to report financial toxicity [25, 28]. Being currently under treatment might be related to more uncertainties regarding the future where financial aspects might play an important role. Also, high OOPPs might be related to financial toxicity during treatment. Although Germany has a universal health coverage, OOPPs during treatment and in aftercare cannot be neglected [14].

A limitation of our analysis is the cross-sectional design which does not allow causal pathways to be established. Additionally, financial difficulties were assessed using a single question scale, which might not cover all aspects related to financial toxicity in cancer patients [7]. Nevertheless, the scale used is part of an internationally validated and accepted questionnaire for measuring quality of life in cancer patients. Changes in the societal financial situation might also impact financial toxicity in patients. Since data was obtained in a relatively short time frame (09/2017 to 02/2019), no significant changes in the societal financial situation occurred during that time frame which could affect the results. Finally, it is also possible that the study population is not fully representative of all sarcoma patients in Germany since participants were mostly recruited from university hospitals or specialized centers. On the other hand, we did evaluate the question of financial toxicity in one of the largest sarcoma cohorts reported in the literature, covering all relevant histological subtypes and treatment groups. With a broad set of sociodemographic and clinical variables available, we were able to identify factors which might have an impact on the chance of reporting financial difficulties that were not addressed in other studies.

## Conclusion

A high proportion of sarcoma patients in Germany report financial difficulties, compared to a general norm population. This impairment appears to be relevant and needs to be addressed by the involved stakeholders, especially since many of the factors associated with financial difficulties in our study were related to income and ability to work. Both treating physicians and patients should be aware of the involved risks and the options regarding the return to a working environment should be discussed with the patients at an early stage. Additional support and consulting opportunities, taking the experience of previous patients into consideration, should be readily available. Furthermore, we believe that policy changes regarding sick leave and the disability pension in Germany should be considered for reducing financial inequality among sarcoma patients.

## Data Availability

The data is not to be deposited but is available on request.

## Code availability

Code is available upon request.
